# Cross-Priming Dendritic Cells Exacerbate Immunopathology After Ischemic Tissue Damage in the Heart

**DOI:** 10.1161/CIRCULATIONAHA.120.044581

**Published:** 2020-12-10

**Authors:** Elvira Forte, Bryant Perkins, Amalia Sintou, Harkaran S. Kalkat, Angelos Papanikolaou, Catherine Jenkins, Mashael Alsubaie, Rasheda A. Chowdhury, Theodore M. Duffy, Daniel A. Skelly, Jane Branca, Mohamed Bellahcene, Michael D. Schneider, Sian E. Harding, Milena B. Furtado, Fu Siong Ng, Muneer G. Hasham, Nadia Rosenthal, Susanne Sattler

**Affiliations:** 1 The Jackson Laboratory, Bar Harbor, ME (E.F., B.P., T.M.D., D.A.S., J.B., M.B.F., M.G.H., N.R.).; 2 National Heart and Lung Institute, Imperial College London, UK (A.S., H.S.K., A.P., C.J., M.A., R.A.C., M.B., M.D.S., S.E.H., F.S.N., N.R., S.S.).; 3 Amgen Biotechnology, Thousand Oaks, CA (M.B.F.).

**Keywords:** autoimmunity, dendritic cells, heart failure, myocardial infarction, myocardial ischemia

## Abstract

**Background:**

Ischemic heart disease is a leading cause of heart failure and despite advanced therapeutic options, morbidity and mortality rates remain high. Although acute inflammation in response to myocardial cell death has been extensively studied, subsequent adaptive immune activity and anti-heart autoimmunity may also contribute to the development of heart failure. After ischemic injury to the myocardium, dendritic cells (DC) respond to cardiomyocyte necrosis, present cardiac antigen to T cells, and potentially initiate a persistent autoimmune response against the heart. Cross-priming DC have the ability to activate both CD4^+^ helper and CD8^+^ cytotoxic T cells in response to necrotic cells and may thus be crucial players in exacerbating autoimmunity targeting the heart. This study investigates a role for cross-priming DC in post–myocardial infarction immunopathology through presentation of self-antigen from necrotic cardiac cells to cytotoxic CD8^+^ T cells.

**Methods:**

We induced type 2 myocardial infarction–like ischemic injury in the heart by treatment with a single high dose of the β-adrenergic agonist isoproterenol. We characterized the DC population in the heart and mediastinal lymph nodes and analyzed long-term cardiac immunopathology and functional decline in wild type and *Clec9a*-depleted mice lacking DC cross-priming function.

**Results:**

A diverse DC population, including cross-priming DC, is present in the heart and activated after ischemic injury. *Clec9a*^−/−^ mice deficient in DC cross-priming are protected from persistent immune-mediated myocardial damage and decline of cardiac function, likely because of dampened activation of cytotoxic CD8^+^ T cells.

**Conclusion:**

Activation of cytotoxic CD8^+^ T cells by cross-priming DC contributes to exacerbation of postischemic inflammatory damage of the myocardium and corresponding decline in cardiac function. Importantly, this provides novel therapeutic targets to prevent postischemic immunopathology and heart failure.

## Abstract 2

Supplemental Digital Content is available in the text.

**Clinical Perspective**

**What Is New?**

- This is the first report implicating the cross-priming function of dendritic cells in immunopathology after type 2 myocardial infarction, including inflammation, fibrosis, and functional decline.
- Myocardial injury leads to local infiltration and activation of cross-priming dendritic cells, which activate cytotoxic T cells that infiltrate the heart.
- Depletion of the dendritic cell cross-priming function inhibits accumulation and activation of cytotoxic T cells and stops myocardial immunopathology and functional decline.

**What Are the Clinical Implications?**

- Cross-priming dendritic cells are activated early as part of the acute immune response after myocardial infarction yet have a long-lasting legacy by triggering activation of a persistent adaptive response in the form of cytotoxic T cells.
- We show that elevated levels of cytotoxic T cells are present in remote areas of human heart failure hearts, where they are highly likely to induce myocardial necrosis.
- With cross-priming, we provide a targetable pathway to prevent activation of T cell cytotoxicity and persistent post–myocardial infarction immunopathology exacerbating heart failure risk.

Heart failure (HF) is a common long term complication of myocardial infarction (MI) affecting up to 62% of MI patients^1^ and >37.7 million people worldwide.^2^ There is no cure and 5-year mortality rates rival those of many cancers.^3^ An MI is defined as cardiac tissue damage attributable to insufficient oxygen supply to parts of the myocardium and is further divided into type 1 MI (T1MI), type 2 MI (T2MI), and myocardial injury.^4,5^ T1MI occurs after disruption of an atherosclerotic plaque leads to a blockade of coronary blood flow, while T2MI is a heterogeneous syndrome where other conditions including hypotension, anemia, respiratory failure, tachycardia, severe hypertension, or sepsis lead to an imbalance between oxygen supply and demand.^6,7^ T2MI is an underrecognized clinical entity with a prevalence of up to 58% of all MI patients.^8^ Notably, these patients have poor short- and long-term outcomes^9^ with a HF risk equal to T1MI patients.^1,10^

A timely immune response to myocardial damage plays a crucial role in endogenous repair processes and tissue maintenance. However, excessive tissue necrosis can lead to persistent inflammation, fibrosis, and a break in immunologic self-tolerance, leading to long lasting adaptive immune autoreactivity.^11–15^ Anti-heart autoantibodies against cardiac epitopes are a well-known clinical phenomenon during HF,^16^ and the pathogenic function of autoreactive T cells after MI has recently been demonstrated.^17,18^ Antigen-specific T and B cells can be long-lived and have been suggested to cause ongoing low-level tissue destruction,^19,20^ hamper regenerative efforts, and exacerbate the development toward heart failure.^21,22^

Dendritic cells (DC) play a central role in triggering autoimmunity. They are considered the most efficient antigen-presenters, linking the innate with the adaptive immune response by processing antigens, presenting them to T cells via major histocompatibility complex (MHC):T cell receptor recognition and defining the resulting T cell functional phenotype depending on inflammatory context.^23^ The DC population has been reported to be involved in the post-MI immune response in rodent T1MI models.^24^ Notably, a recent study using highly specific depletion of DC via *Zbtb46* elegantly showed reduced post-MI infarct size, improved systolic function, and reduced total T cell numbers in the ischemic tissue.^25^

Importantly, the total DC population is highly heterogenous. While classic antigen presentation is mediated by the interaction between antigenic peptide-bound MHC II molecules with their cognate T cell receptor on CD4^+^ helper T cells,^26^ a subpopulation of DC (classical DC1) has the distinct ability to present antigen to both CD4^+^ helper and CD8^+^ cytotoxic T cells.^27^ This “cross-priming” activity is a powerful boost for adaptive immune responses and has been exploited for improved anticancer vaccines.^28^ However, in a situation of necrotic tissue injury such as cardiomyocyte cell death, DC cross-priming of cardiac antigen to cytotoxic CD8^+^ T cells may exacerbate long-term autoimmune-mediated tissue damage.

To explore the role of the cross-priming subset of classical DC (cDC1) in immune-mediated myocardial deterioration after ischemic tissue damage, we show here that cross-priming DC are present in the heart and activated after ischemic injury. Genetic depletion of the C-type lectin-like receptor gene *Clec9a*, which enables DC cross-priming, decreases long-term myocardial immunopathology, protects from decline of cardiac function, and suppresses the activation of cytotoxic CD8^+^ T cells.

## Materials and Methods

An expanded methods section is available in the Data Supplement.

### Data Availability

The accession number for the single cell sequencing data reported in this paper is ArrayExpress E-MTAB-7895.^29^ All other data, methods, R scripts, and materials that support the findings of this study are available from the authors on request.

### Mice

All animal procedures were approved by the Imperial College Governance Board for Animal Research and the Home Office (UK) and The Jackson Laboratory Institutional Animal Care and Use Committee, respectively. Mouse strains used in this study were B6(Cg)-*Clec9a*^*tm1.1Crs*^/J (CLEC9a^−/−)^, B6.129S2-Cd8a^tm1Mak/^J (*CD8*^−/−^), and C57BL/6J (wild type [WT]). T1MI was induced by permanent ligation of the left anterior descending artery as previously described^30^ in male and female mice 10 to 12 weeks of age. T2MI was induced by a single intraperitoneal injection at a dose of 160 mg/kg isoproterenol HCL (Sigma-Aldrich, St. Louis, MO), as described previously.^31^ Monitoring of cardiac function was performed by transthoracic echocardiography by a blinded operator using a high-frequency ultrasound system Vevo 770 (VisualSonics Inc., Toronto, Canada) with a 30-MHz linear transducer.

### Human Tissue

All work was carried out under the Human Tissue Act 2004 and conformed with the principles of the Declaration of Helsinki. Tissue and clinical data were fully anonymized before being handed to researchers. Human left ventricular (LV) tissue was obtained from hearts of end-stage heart failure patients removed during transplant surgery (Research Ethics Committee approval: 09/H0504/104+5; Biobank approval number: NP001-06-2015). Donor hearts unsuitable for transplantation were used as controls (Research Ethics Committee approval: 16/LO/1568). Informed consent was obtained from patients or next of kin. For detection of CD3^+^ and CD8^+^ T cells in heart failure hearts, frozen sections of human heart tissue were stained with mouse anti-human CD3 (clone UCHT1 at 1:100), and mouse anti-human CD8 (clone HIT8a at 1:100) with Alexa Fluor 488 goat anti-mouse IgG (poly4053 at 1:500; all purchased from BioLegend, London, UK).

### Single Cell Sequencing

Single cell RNA sequencing of MI hearts was performed as part of a previous study^29^ using the 10× Chromium platform with v2 chemistry and analyzed using Seurat software package version 3.0^32^ in R version 3.6.2.^33^ All sequencing data are available in the Array Express repository (accession number EMTAB-7895).

### Histology and Immunofluorescence Staining

Hearts were excised after perfusion and processed for standard hematoxylin and eosin and Picrosirius red histological stains. Semiquantitative scoring was performed, as previously established.^34^ For CD8 immunohistochemistry, sections were stained with rat anti-mouse CD8 (14-0808-80 at 1:1000; Invitrogen; Thermo Fisher Scientific, Rochford, UK) and rabbit anti-rat horseradish peroxidase (AI-4001 at 1:50; Vector Laboratories, Peterborough, UK) with hematoxylin as nuclei counterstain. Image analysis and acquisition was performed using a Hamamatsu NanoZoomer 2.0 slide scanner (Hamamatsu, San Jose, CA), a LMD7000 microscope (Leica Microsystems, Milton Keynes, UK), NDP.view2 Plus Image viewing software (Hamamatsu) and public domain software ImageJ (http://rsb.info.nih.gov; National Institutes of Health),^35^ and QuPath open source bioimage analysis software (https://qupath.readthedocs.io/en/latest/index.html#).^36^

### Langendorff Perfusion and Optical Mapping

Experiments were performed as previously described.^37,38^ Briefly, explanted hearts were perfused and superfused with modified Tyrode’s solution (in mM: 130 NaCl, 5.4 KCl, 0.3 NaH_2_PO_4_, 1 MgCl_2_, 10 HEPES, 18 CaCl_2_, and 10 D-Glucose, [pH 7.4]) ECGs were recorded during all experiments using LabChart 8 software in conjunction with Powerlab and BioAmp systems (ADInstruments Pty Ltd, UK) and programmed electric stimulation protocols were carried out via the pacing electrode at the left ventricle using a stimulator (MicroPace, USA). For optical mapping of transmembrane voltage, an excitation-contraction uncoupler, blebbistatin (5 μM), and voltage sensitive dye, Di-4-ANNEPS (5 μM) were administered to the heart and signals recorded using complimentary metal-oxide semiconductor cameras (RedShirtImaging LLC, USA). ECG recordings and optical recordings were analyzed using methods previously described.^37,38^

### Cardiac Troponin I ELISA

A mouse cardiac troponin I ELISA Kit (MyBioSource, San Diego, CA) was used to assess cardiac troponin I levels in post-MI serum according to the manufacturer’s instructions.

### Tissue Digestion and Flow Cytometry

To generate single-cell suspensions for flow cytometry, a modified digestion protocol was used as previously described.^39^ Cell suspensions were stained with a previously established flow cytometry panel.^31^ Samples were acquired using a BD FACSymphony A5 (Becton Dickinson) and analyzed using FlowJo 10.2 (Treestar, Ashland, OR) software (www.flowjo.com).

### Experimental Planning and Statistical Analysis

Experimental design of in vivo mouse studies was guided by the recommendations of the National Centre for the Replacement, Refinement and Reduction of Animals in Research.^40^ Statistical analysis was performed using SPSS or GraphPad Prism 6 and data were presented as mean±SEM throughout. Comparison between 2 groups was performed using Student *t* test. Comparison between multiple experimental groups was performed using 1- or 2-way ANOVA with multiple comparisons post hoc tests to obtain multiplicity-adjusted *P* values. Differences were considered significant at *P*<0.05.

## Results

### Ischemic Injury to the Heart Activates Dynamic Changes in the Myocardial DC Population

**Figure 1.**
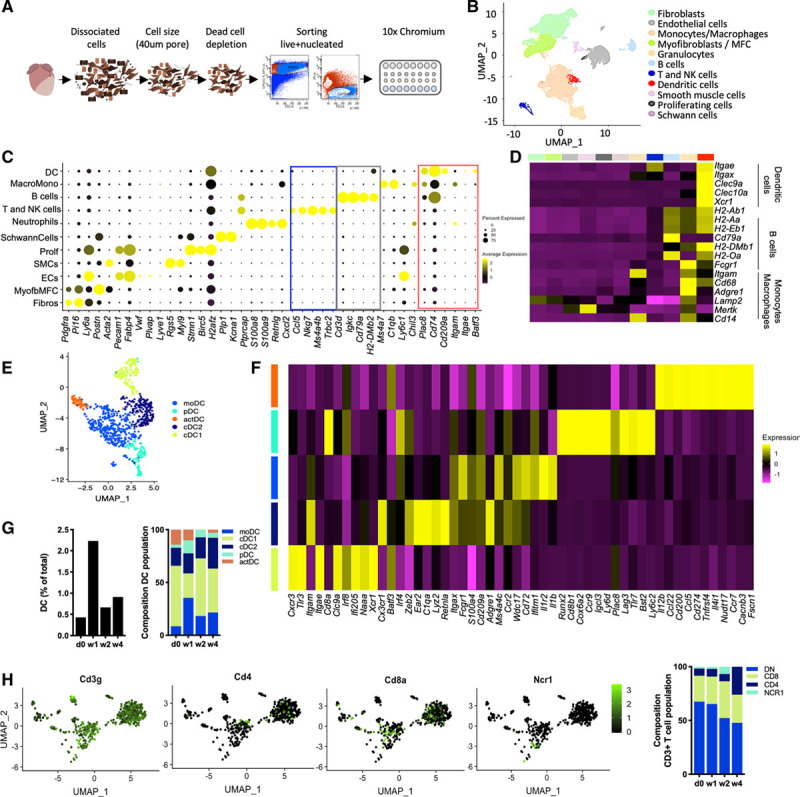
**DC and T cells after ischemic injury in the heart.** **A**, Experimental approach used to obtain the single cell RNA sequencing dataset. Mouse cardiac interstitial cells were isolated by mechanical and enzymatic dissociation of adult mouse cardiac ventricular tissue at homeostasis and over time post–myocardial infarction. Single, live, nucleated interstitial cells were used for the 10× Chromium analysis; 51 687 were captured and sequenced. **B**, UMAP plots highlighting DC in orange, and T and NK cells in blue. **C**, Dot plot showing top marker genes for each lineage. The size scale is proportional to the percentage of expressing cells; color scale indicates average expression intensity. **D**, Heatmap comparing the expression level of B cells/DC/macrophage marker genes across all populations. **E**, UMAP showing the DC subclusters (897 cells). **F**, Heatmap of top marker genes for each DC subpopulation. **G**, Quantification of DC among total cells and relative frequency of DC subpopulations at homeostasis and different time points post–myocardial infarction. **H**, Feature plots showing the expression of *Cd3g, Cd4, Cd8a* and *Ncr1* in a subset of cells defined by *Cd3g* expression (1040 cells). **I**, Quantification of *Cd4+, Cd8a+*, *Ncr1*+ and DN cells within the *Cd3g*+ fraction of the NK and T cell cluster. actDC indicates activated DC; cDC1, classical DC1; cDC2, classical DC2; DC, dendritic cells; DN, double negative; EC, endothelial cells; MFC, matrifibrocytes; moDC, monocytes/monocyte-derived; NK, natural killer; pDC, plasmacytoid DC; and UMAP, uniform manifold approximation and projection.

Single cell RNA sequencing identified an expectedly heterogenous DC population in the mouse heart, both at baseline and over time after myocardial ischemic injury. Myocardial injury was induced by surgical ligation of the left anterior descending artery and single cell RNA sequencing was performed on 51 687 single, live, nucleated interstitial (noncardiomyocyte) cells, using the 10× Chromium v2 technology^41^ as described previously^29^ (Figure 1A). Unbiased clustering defined a distinct population of *H2-Ab1*^+^, *Cd209a*^+^ cells as DC (Figure 1B and 1C). This classification was confirmed by comparing the expression levels of bona fide DC markers and markers shared with other antigen-presenting cells (B cells, monocyte/macrophages) across all cell populations (Figure 1D). Unbiased clustering further split the DC population into 5 subclusters (Figure 1E). These included CD103 (*Itgae*)^−^ CD11b (*Itgam*)^+^ classical DC1 (cDC1), CD103 (*Itgae*)^+^ CD11b (*Itgam*)^−^ cDC2, monocytes/monocyte-derived DC, plasmacytoid DC, and a small population of “activated DC.” Activated DC are characterized by markers upregulated in response to stimulation, such as *Cacnb3*,^42^ *Ccr7, Il12b* involved in priming Th1 and cytotoxic T cell responses, and features of migratory DC (eg, *Fscn-1, Cd200, Cd274*^43^; Figure 1F). Of particular interest in a setting of necrotic tissue injury was the presence of cDC1 cells, which expressed genes involved in cross-presentation of endogenous antigens including *Xcr1* and *Clec9a* (Figure 1F). As previously shown in left anterior descending artery ligation-induced myocardial necrosis,^44^ the number of DC increased after MI reaching a striking 2% of the total noncardiomyocyte cell population 1 week after MI before returning to near baseline levels. cDC1 constituted the majority of DC at baseline (57%) and again after week 2 (54%) once acute infiltration of monocytes/monocyte-derived DC had resolved (Figure 1G).

Interestingly, we also observed a predominance of cytotoxic *Cd8*^+^ over *Cd4*^+^ helper T cells and a notable proportion of *Cd8*^−^*Cd4*^−^ double negative T cells within the *Cd3*^+^ cell population in the T cells cluster (Figure 1H and 1I). These were negative for *Ncr1*, which excludes an NKT phenotype (Figure 1I). The biological significance of these cells is currently under investigation. These observations prompted us to further study DC activation after ischemic injury in the heart, and potential effects of cross-priming DC on postischemic autoimmune-mediated damage and the cytotoxic CD8^+^ T cell population.

### Ischemic Injury Leads to an Increase in Activated and XCR1^+^ Cross-Priming DC in the Myocardium and the Heart-Draining Mediastinal Lymph Nodes

**Figure 2.**
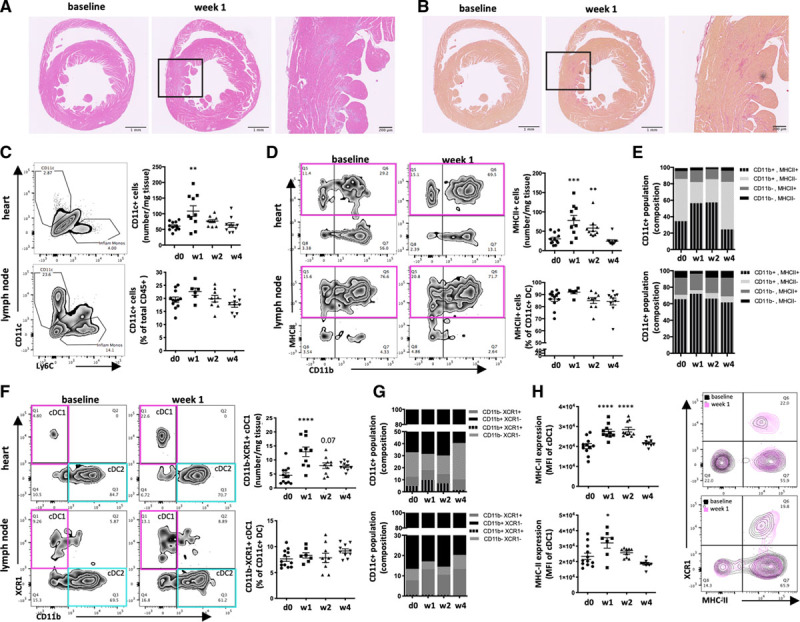
**Type 2 myocardial infarction–like myocardial injury activates the DC population in the heart and the heart-draining lymph nodes.** C57BL/6J mice were treated with 160 mg/kg isoproterenol to induce T2MI-like myocardial injury and flow cytometry was performed on a single cell preparation of the heart and mediastinal lymph nodes. **A** and **B**, Example micrographs (modified from Hasham et al^34^) of isoproterenol-induced myocardial histopathology as assessed by hematoxylin and eosin staining for immunopathology (**A**) and Picrosirius red staining for fibrosis (**B**). **C**, Flow cytometry contour plots showing the gating for CD11c^+^ population among total CD45^+^ and corresponding quantification of cells/mg tissue (heart) or percent of total CD45^+^ cells (lymph node). **D**, Representative contour plots of CD11b and MHC-II staining in the CD11c^+^ population of hearts and lymph nodes of isoproterenol-treated versus control mice and corresponding quantification of CD11c^+^MHC-II^+^ cells/mg tissue (heart) or as percent of total CD11c^+^ cells (lymph node). **E**, Composition of the CD11c^+^ cell population in heart and lymph nodes based on CD11b and MHC-II expression. **F**, Representative contour blots of CD11b and XCR1 staining within the CD11c^+^ population of hearts and lymph nodes of isoproterenol-treated versus control mice and corresponding quantification of CD11b^−^XCR1^+^ cells/mg tissue (heart) or as percent of total CD11c^+^ cells (lymph node). **G**, Composition of the CD11c^+^ cell population in heart and lymph nodes based on CD11b and XCR1 expression. **H**, Levels of MHC-II expression (MFI) in CD11b^−^XCR1^+^ cDC1 cells in heart and lymph node and corresponding representative contour blots. Symbols represent individual mice. Error bars show mean±SEM; *P<0.05; **P<0.001; ***P<0.0001; ****P<0.00001 (1-way ANOVA with Dunnett multiple comparisons post hoc test comparing each time point to baseline, multiplicity-adjusted *P* values). cDC1, classical dendritic cell 1; DC, dendritic cells; MFI, mean fluorescence intensity; and MHC, major histocompatibility complex.

To test the generality of the DC response to cardiac insult, T2MI-like necrotic lesions in the myocardium were induced by a single intraperitoneal injection of isoproterenol. This treatment regime was previously optimized for C57Bl/6J mice^31^ and induced myocardial histopathology reminiscent of T2MI, including inflammatory lesions (Figure 2A) and replacement fibrosis (Figure 2B). Single cell suspensions obtained from digested ventricular tissue and heart-draining mediastinal lymph nodes were analyzed for the presence of cDC at baseline, weeks 1, 2, and 4 after injury. cDC were defined by flow cytometry as CD45^+^CD3^−^CD19^−^Ly6G^−^Ly6C^−^CD11c^+^ (Figure I in the Data Supplement). Consistent with the above single cell RNAseq results, the total number of CD11c^+^Ly6C^−^ cDC cells per milligram ventricular tissue was increased in the heart 1 week after injection and subsequently returned to baseline numbers. In contrast, the relative proportions of lymph node cDC did not change significantly (Figure 2C). MHC-II expression on DC is an indicator for their maturation state and antigen presenting capacity.^45,46^ Comparable with total cDC numbers, the number of MHC-II expressing cells also increased quickly after isoproterenol injection (Figure 2D) indicating a heightened maturation state and increased capacity for antigen presentation within the cardiac cDC population (Figure 2E). Again, no significant change in cDC composition was observed in mediastinal lymph nodes of isoproterenol-treated mice.

Subsequently, we used XCR1 as marker to discriminate cDC1 (CD11b^−^XCR1^+^) from cDC2 (CD11b^+^XCR1^−^) among total cDC, as XCR1 is expressed on both peripheral and lymphatic tissue cDC1 but is absent from cDC2.^47^ In the heart, the CD11c^+^Ly6c^−^ cDC population consisted of CD11b^+^XCR1^−^ cDC2 and CD11b^−^XCR1^+^ cDC1 (Figure 2F). A small proportion of CD11b^−^XCR1^−^ cells was observed which did not change over time. cDC1 numbers increased significantly in the heart (numbers per milligram tissue) but not in the lymph node (percent of total cDC; Figure 2G). Importantly, in both the heart and the lymph node, cDC1 cells upregulated MHC-II expression indicating an increased maturation state and higher antigen-presentation ability. Importantly, besides an overall increase in the number of MHC-II positive DC, the cDC1 subpopulation (CD11c^+^CD11b^−^XCR1^+^) also upregulated antigen-presentation function as seen via an increase in MHC-II mean fluorescent intensity levels (Figure 2H).

These results confirm that the cross-priming cDC1 population is activated in response to myocardial tissue injury, which may provide the critical first step for induction of cardiac immunopathology.

### Blockade of DC Cross-Priming by Genetic Depletion of *Clec9a* Allows More Efficient Resolution of Postischemic Inflammation and Immunopathology

To confirm a role of DC cross-priming in the immune response to cardiac injury, we made use of mice lacking the gene encoding CLEC9A (C-type lectin-like receptor 9A)/DNGR-1 (dendritic cell natural killer lectin group receptor 1)/CD370, a receptor essential for processing antigens from necrotic cells for cross-priming of cytotoxic CD8^+^ T cells.^48^ *Clec9a*^−/−^ mice express green-fluorescent protein under the control of the *Clec9a* promotor. This confirmed the specificity of CLEC9A expression for XCR1+ DC (Figure II in the Data Supplement). While granulocytes, and in particular a small proportion of eosinophils, appear to express some CLEC9A, the vast majority and by far highest expression was detected in cDC1.

Myocardial damage induced by a single dose of isoproterenol was assessed by semiquantitative scoring and quantification of immunopathology/immune cell infiltration and collagen deposition as described previously.^34^ Results were consistent between high throughput semiquantitative scores, which allow reliable assessment of the disruption of healthy cardiac histomorphology attributable to immune cell clusters and inflammation-associated edema, and quantitative image analysis, which affords accurate cell/nuclei counts and measures of collagen area (Figure III in the Data Supplement). Thus, subsequent analysis was performed using semiquantitative scoring.

**Figure 3.**
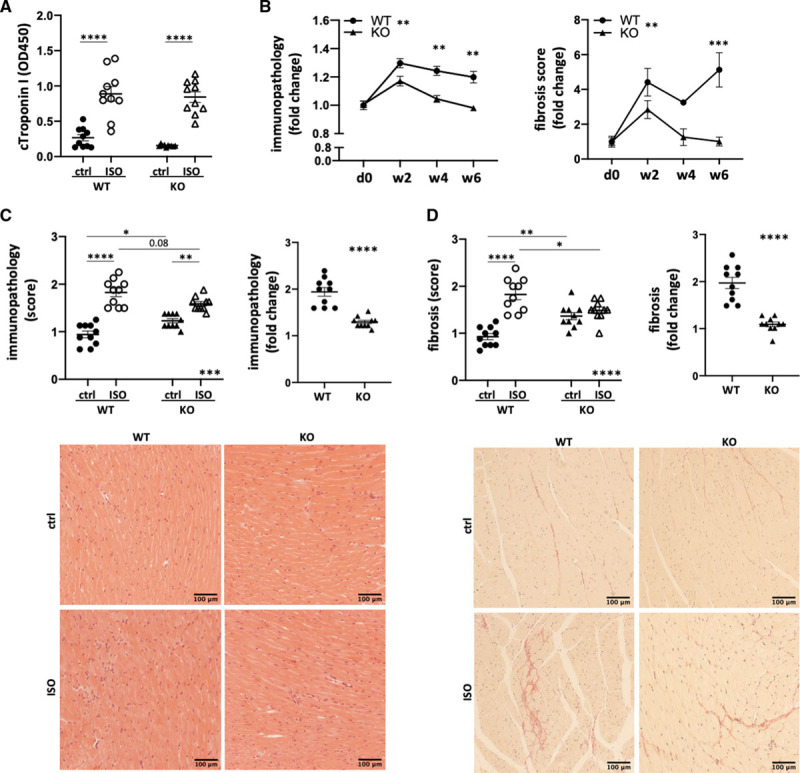
**Blockade of DC cross-priming by genetic depletion of *Clec9a* protects mice from immunopathology after ischemic injury.** C57BL/6J (WT) and *Clec9a*^−/−^C57BL/6J (KO) mice were treated with 160 mg/kg isoproterenol to induce T2MI-like injury and hearts were isolated for histology and immunohistochemistry. **A**, cTroponin I levels in serum of WT and KO 24 h after isoproterenol challenge. **B**, Mononuclear infiltrate/immunopathology and fibrosis in the myocardium of isoproterenol-treated WT and KO mice as assessed by quantification of nuclei in hematoxylin and eosin–stained heart sections over time (day 0, week 2, week 4) after injury. **C**, Semiquantitative immunopathology score and corresponding changes per mouse strain in myocardial sections of isoproterenol-treated WT and KO mice as assessed by hematoxylin and eosin–stained histology 4 weeks after injury. **C**, **lower**, Example micrographs per experimental group. **D**, Fibrotic area and corresponding changes per mouse strain in myocardial sections of isoproterenol-treated WT and KO mice as assessed by Picrosirius red–stained histology 4 weeks after injury. **D**, **lower**, Example micrographs per experimental group. Symbols represent individual mice. Error bars show mean±SEM; *P<0.05; **P<0.001; ***P<0.0001; ****P<0.00001 (2-way ANOVA with Sidak multiple comparisons post hoc test; 2-tailed Student *t* test for fold-change data [**C** and **D**] multiplicity-adjusted *P* values). Ctrl indicates control; DC, dendritic cell; ISO, isoproterenol; KO, knockout; and WT, wild type.

Both Clec9a^−/−^ and WT mice reacted to isoproterenol treatment with an equal increase in serum troponin levels 24 hours after injection (Figure 3A), mononuclear infiltration, and fibrosis in the myocardium (Figure 3B). Strikingly, however, myocardial histology of isoproterenol-treated *Clec9a*^−/−^ mice was indistinguishable from untreated controls by week 4, while immunopathology and fibrosis persisted until week 6 in WT mice (Figure 3B). A subtle but nonsignificant difference at early time points (week 2) suggested protection from acute inflammatory damage because of depletion of *Clec9a*. This has been shown previously in acute pancreatitis and systemic candidiasis and has been explained by a dampening effect on neutrophil-mediated tissue immunopathology.^49^ Notably however, the frequency of CD11b^+^ myeloid cells, CD11b^+^Ly6g^+^ neutrophils, and CD11c^+^ DC among total leukocytes in the myocardium and lymph nodes, both at baseline and at week 1 after isoproterenol injection, was unaffected by *Clec9a* depletion (Figure IV in the Data Supplement).

As the focus of this study was long term immunopathology, a more detailed analysis of histopathology was performed at later time points postinjection. *Clec9a*^−/−^ depletion resulted in dampened immunopathology (Figure 3C) and fibrosis (Figure 3D) in mice. We also observed a mild distortion of healthy tissue morphology in these baseline *Clec9a*^−/−^ mouse hearts, presumably because of the loss of a peripheral tolerance mechanism^50,51^ (Figure 3C and 3D). However, a thorough investigation of cardiac histopathology (Figure V in the Data Supplement), as well as characterization of the immune cell population in myocardium and mediastinal lymph nodes, did not reveal any significant differences between healthy adult knockout (*Clec9a*^−/−^), heterozygous (*Clec9a*^+/−^), and WT (*Clec9a*^+/+^) mice (Figure V in the Data Supplement) at 10 weeks of age. Echocardiography and hematology data obtained from the International Mouse Phenotyping Consortium (available at www.mousephenotype.org) further confirms that young adult *Clec9a*^−/−^ depleted mice (Clec9a^tm1b(KOMP)Wtsi^) do not vary significantly from WT controls.^52^ This shows the necessity of a respective baseline control for each experimental group to control for batch effects, influences of animal age, or subtle variation of baseline phenotypes between C57BL/6J and *Clec9a*^−/−^ strains. Both sexes showed comparable responses, despite a small additional protective effect in females (Figure VI in the Data Supplement).

In summary, inflammation and fibrosis induced by myocardial damage are dampened by blockade of DC cross-priming, presumably because of faster resolution of the inflammatory process despite a largely comparable degree of initial injury.

### Blockade of DC Cross-Priming Protects From Cardiac Adverse Remodeling and Functional Decline After Ischemic Injury

**Figure 4.**
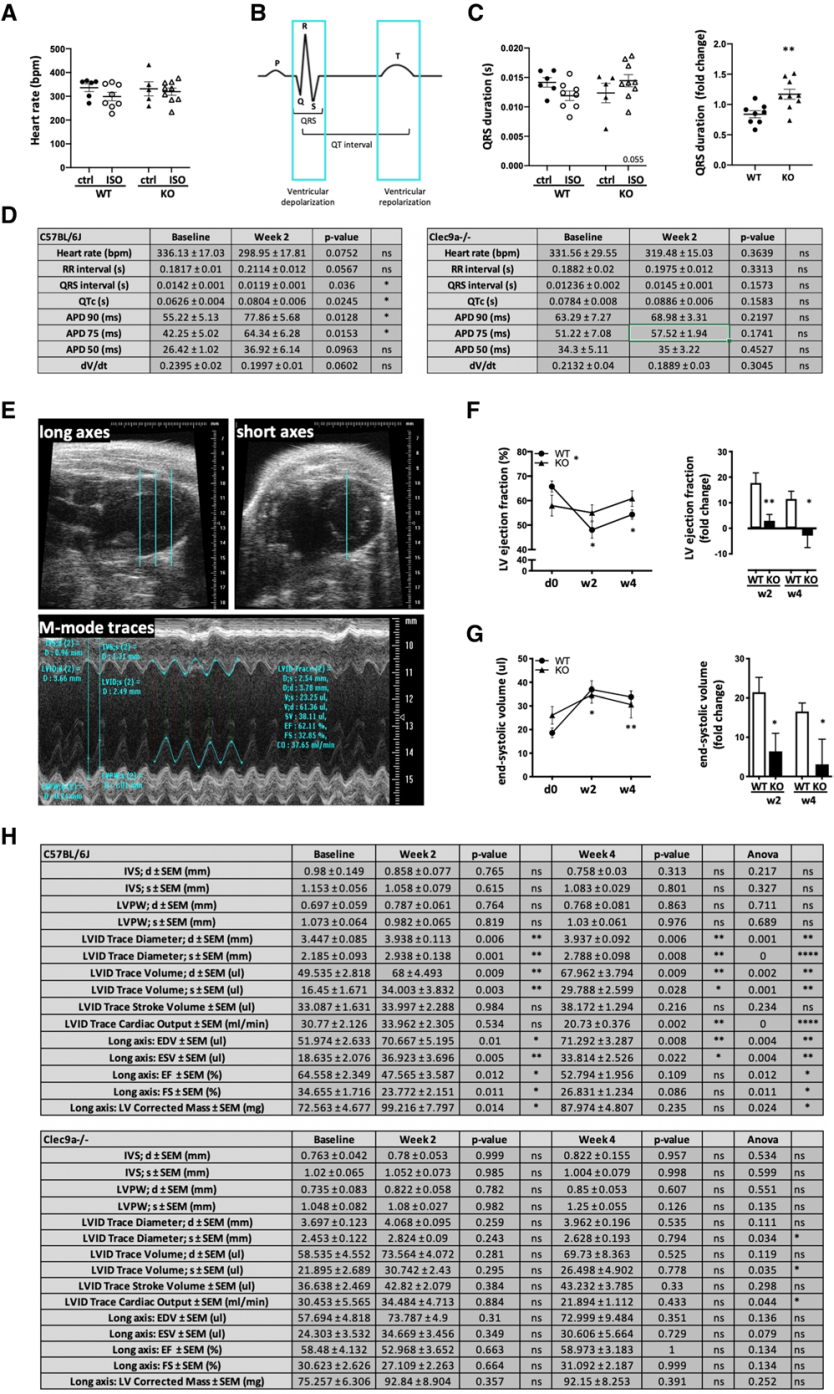
**Blockade of DC cross-priming protects mice from adverse remodeling and functional decline after ischemic injury.** C57BL/6J (WT) and *Clec9a*^−/−^ C57BL/6J (KO) mice were treated with 160 mg/kg isoproterenol to induce T2MI-like injury and heart function was assessed by electrocardiography, optical mapping and transthoracic echocardiography. **A**, Heart rate of isoproterenol-treated WT and KO mice 2 weeks after challenge. **B**, Schematic showing features of electrocardiography traces. **C**, Quantification of QRS duration in electrocardiography traces of isoproterenol-treated WT and KO mice 2 weeks after challenge and corresponding fold change of QRS duration in both strains. Symbols represent individual mice. **D**, Summary of parameters obtained by electrocardiography and optical mapping. **E**, Parasternal long- and short-axis echocardiography B-mode view (blue lines in long and short axes) showing levels of M-mode traces (base, mid, apex) and obtained measurements. **F**, LV global function in isoproterenol-treated WT and KO mice as assessed by % ejection fraction (**left**) and fold decrease from baseline (right). **G**, LV dilation in isoproterenol-treated WT and KO mice as assessed by LV end-systolic volume (left) and fold increase from baseline (right). **H**, Summary of echocardiography parameters obtained by echocardiography M-mode traces from both the parasternal long- and short-axis view; n=6/group. Error bars show mean±SEM; *P<0.05; **P<0.001; ***P<0.0001; 2-way ANOVA with Sidak multiple comparisons post hoc test (**A**, **C**, **F**, **G**); 2-tailed Student *t* test (**C** and **D**); repeat measures 2-way ANOVA with Sidak multiple comparisons post hoc test (**F** and **G**); 1-way ANOVA with Dunnett multiple comparisons post hoc test (**H**); multiplicity-adjusted *P* values. APD, action potential duration; ctrl, control; d, diastole; d0, day 0; dV/dt, rate of action potential rise; EDV, end diastolic volume; EF, ejection fraction; ESV; end systolic volume; FS, fractional shortening; ISO, isoproterenol; IVS, interventricular septum (thickness); KO, knockout; LV, left ventricular; LVID, left ventricular internal dimension; LVPW, left ventricular posterior wall (thickness); s, systole; w2, week 2; w4, week 4; and WT, wild type.

To determine whether faster resolution of immunopathology in the *CLEC9a*^−/−^ mice affects cardiac morphology and function, we analyzed ECG parameters and echocardiography in isoproterenol-treated *Clec9a*^−/−^ and WT mice 2 and 4 weeks after injection, respectively (Figure 4). While heart rates remained consistent (Figure 4A), *Clec9a*^−/−^ and WT mice showed different functional responses to isoproterenol challenge. Quantification of ECG traces (Figure 4B, scheme) revealed a difference in isoproterenol-induced changes in QRS duration, an indicator of ventricular depolarization, between WT and Clec9a^−/−^ mice (Figure 4C). While QRS duration appeared to decrease in WT mice, a subtle increase was observed in *Clec9a*^−/−^ mice. A summary of all ECG and optical mapping parameters tested is shown in Figure 4D.

Analysis of systolic function by parasternal echocardiography was performed by analyzing M-mode traces of 3 levels (base, mid/papillary muscle level, apex) in both long and short axes view (Figure 4E, scheme). This showed a significant drop in LV ejection fraction (EF; Figure 4F) which correlated with a clear dilation phenotype with increased LV volumes (Figure 4G) in WT mice. Dilation was detectable but mild in *Clec9a*^−/−^ mice and did not translate into significantly decreased functional decline (Figure 4F and 4G). Thus, while the isoproterenol-induced decrease in EF, as well as increase in LV end-diastolic LV dilation, persisted in WT mice, *Clec9a*^−/−^ mice largely recovered normal cardiac function. Comprehensive echocardiography parameters are shown in Figure 4H.

In summary, blockade of DC cross-priming function not only affects histopathology after myocardial injury, but also impacts on function as it prevents adverse remodeling and corresponding functional decline of the heart.

### Blockade of DC Cross-Priming Function Protects *Clec9a*^−/−^ Mice From Cytotoxic CD8^+^ T Cell Activation

**Figure 5.**
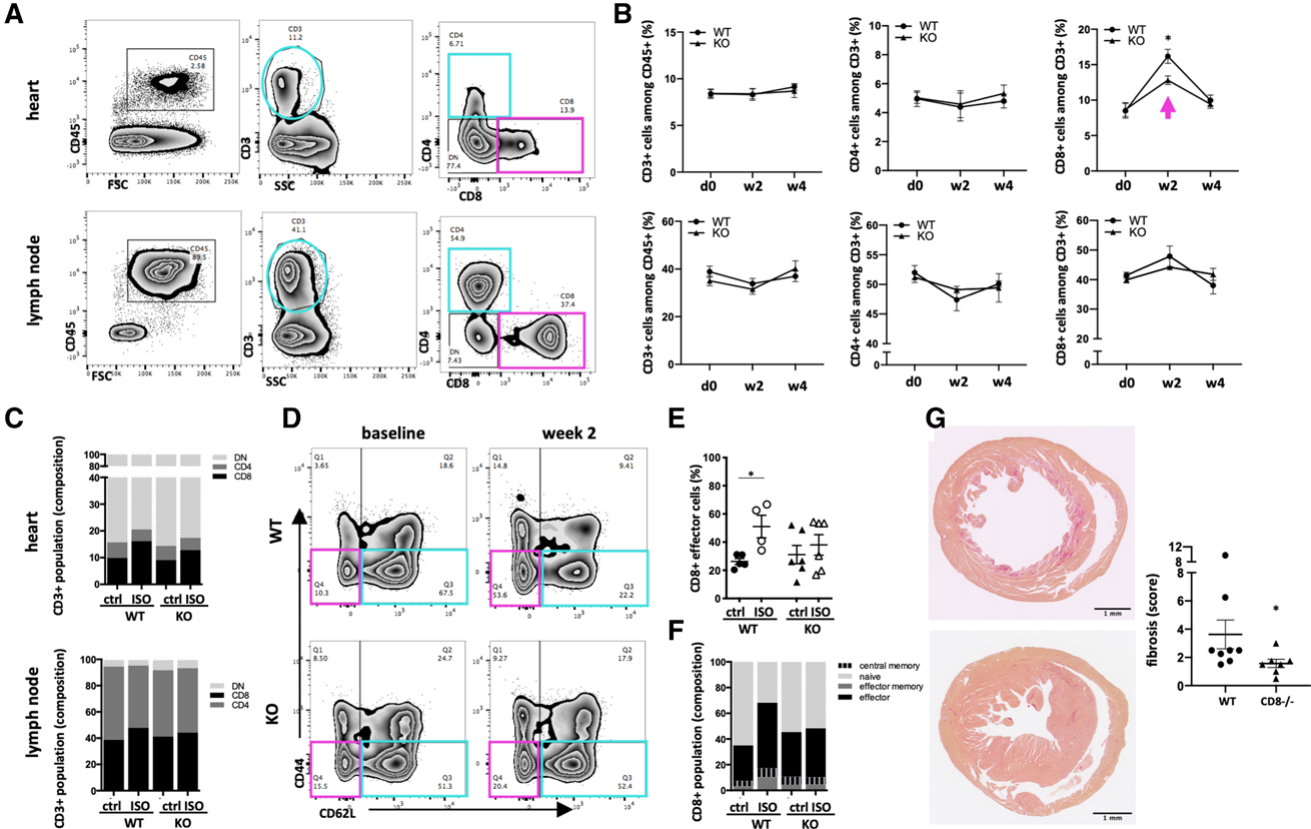
**Protection of *Clec9a*^−/−^ mice after ischemic injury is due to a halt in activation of CD8^+^ T cells.** C57BL/6J (WT) and *Clec9a*^−/−^ C57BL/6J (KO) mice were treated with 160 mg/kg isoproterenol to induce T2MI-like injury and flow cytometry was performed on a single cell preparation of the heart and mediastinal lymph nodes. Isoproterenol-treated *Cd8*^−/−^ mice were used for histopathological assessment of cardiac immunopathology. **A**, Flow cytometry contour blot showing the gating for the T cell population among total CD45^+^ and (**B**) corresponding quantification of CD3^+^, CD4^+^ and CD8^+^ cells in the heart and lymph node; n=4 to 7/group. **C**, Composition of the CD3^+^ cell population in heart and lymph nodes based on CD4 and CD8 expression. **D**, Representative contour blots of CD62L and CD44 staining in the CD8^+^ population of mediastinal lymph nodes of isoproterenol-treated versus control mice. **E**, Quantification of CD62L^−^CD44^+^ effector cells among total CD8^+^ cells in mediastinal lymph nodes. **F**, Composition of the CD8^+^ cell population in lymph nodes based on CD62L and CD44 expression. **G**, Micrographs of Picrosirius red–stained cardiac sections of isoproterenol-treated WT and *Cd8*^−/−^ mice and corresponding semiquantitative fibrosis score. Symbols represent individual mice. Error bars show mean±SEM; *P<0.05; 2-way ANOVA with Sidak multiple comparisons post hoc test (**B** and **E**); 2-tailed Student *t* test (**G**); multiplicity-adjusted *P* values. ctrl indicates control; KO, knockout; ISO, isoproterenol; and WT, wild type.

To elucidate the underlying immunologic mechanism conveying protection from immune-mediated damage in *Clec9a*^−/−^ mice, we performed flow cytometry on the T cell population in the heart and heart-draining mediastinal lymph nodes 2 and 4 weeks after isoproterenol-induced ischemic cardiac injury (Figure 5A, gating).

While the relative contribution of total CD3^+^ and CD3^+^CD4^+^ T cells in the heart and mediastinal lymph nodes remains constant (Figure 5B), a significant shift toward cytotoxic CD8^+^ T cells in the hearts and lymph nodes of WT mice contrasted with an unchanged T cell population in *Clec9a*^−/−^ mice (Figure 5B and 5C). Importantly, as previously suggested,^17^ depletion of CD8^+^ T cells protected mice from immune-mediated myocardial damage, and another recent study confirms pathogenic effects of CD8^+^ T cells after MI.^18^ Consistent with single cell sequencing data above (Figure 1H and 1I), a significant proportion of CD3^+^ cells in the heart expressed neither CD4^+^ nor CD8^+^ (Figure 5C). The biological significance of this finding is currently under investigation.

Importantly, mediastinal lymph node CD8^+^ T cells in WT mice displayed an activated phenotype with downregulated cell adhesion molecule L-selectin/CD62L. The proportion of CD62L^−^CD44^−^ effector cells increased, while CD62L^+^CD44^−^ naïve cells decreased. In addition, a shift from CD62L^+^CD44^+^ central memory to CD62L^−^CD44^+^ effector memory was observed (Figure 5D). In contrast, the activation state of lymph node CD8^+^ T cells in *Clec9a*^−/−^ mice remained at baseline levels (Figure 5E and 5F). This was most prominent 2 weeks after isoproterenol treatment (Figure VII in the Data Supplement). To confirm a role of CD8^+^ T cells, T2MI was induced in WT and *Cd8*^−/−^ mice lacking all functional CD8^+^ cytotoxic T cells. Histopathologic analysis of the hearts was performed 4 weeks after treatment and fibrosis in *Cd8*^−/−^ mice was decreased in comparison to WT control mice (Figure 5G). Thus, blockade of DC cross-priming ability prevents the activation of cytotoxic CD8^+^ T cells, which enhances myocardial immunopathology.

### CD8^+^ T Cell Numbers Are Elevated in Human HF Tissue

**Figure 6.**
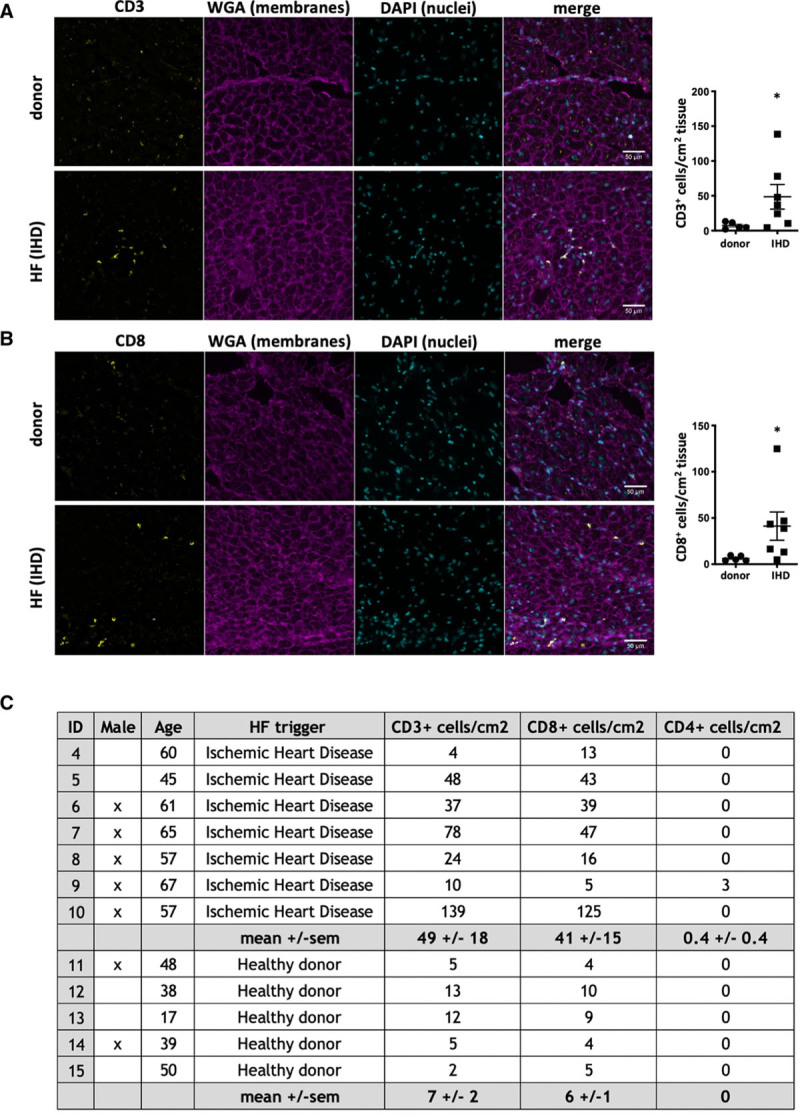
**CD3^+^ and CD8^+^ T cell numbers are elevated in the myocardium of human HF patients.** Human LV tissue was obtained from end-stage HF patients during transplant surgery. Control samples for comparison were obtained from organ donor hearts assessed to be unsuitable for donation. Representative images and corresponding count of the number of CD3^+^ (**A**) and CD8^+^ (**B**) T cells/cm^2^ heart tissue section. **C**, Patient population; n=5 (donors), 7 (ischemic heart disease). Symbols represent individual patients. Error bars show mean±SEM; *P<0.05; **P<0.001; ***P<0.0001 (1-tailed Student *t* test with Welch correction). DAPI indicates 4′,6-diamidino-2-phenylindole; HF, heart failure; IHD, ischemic heart disease; and WGA, wheat germ agglutinin.

To extend these findings to human disease, LV sections were prepared from end-stage human HF hearts obtained during transplant surgery. Remote LV areas with macroscopically healthy appearance were chosen to probe T cell infiltration into the remote myocardium. Control sections were prepared from donor hearts deemed unsuitable for transplantation. Increased numbers of total CD3^+^ T cells (Figure 6A) and CD8^+^ cytotoxic T cells (Figure 6B) were detected in human HF tissue with more CD3^+^ and CD8^+^ cells in 5 out of 7 (71%) ischemic HF patients compared with donors, confirming the relevance of myocardial CD8^+^ T cells in human HF. These results also indicate that increased myocardial T cell numbers are not a simple bystander effect of total immune cell infiltration into damaged tissue, but active infiltration into the otherwise healthy myocardium. Donors were 3 females, 2 males (38±13 years). HF patients were 2 female and 5 male individuals (59±7 years) with end-stage HF attributable to ischemic heart disease after MI (Figure 6C). We also included 3 patients (2 females and 1 male; 33±16 years) with HF attributable to previous myocarditis for a comparison with heart disease of an established immunologic etiology (Figure VIII in the Data Supplement).

### Accumulation of Cytotoxic CD8^+^ T Cells Is Reduced in Both Scarred and Remote Myocardium in *Clec9a*^−^^/−^ Mice

**Figure 7.**
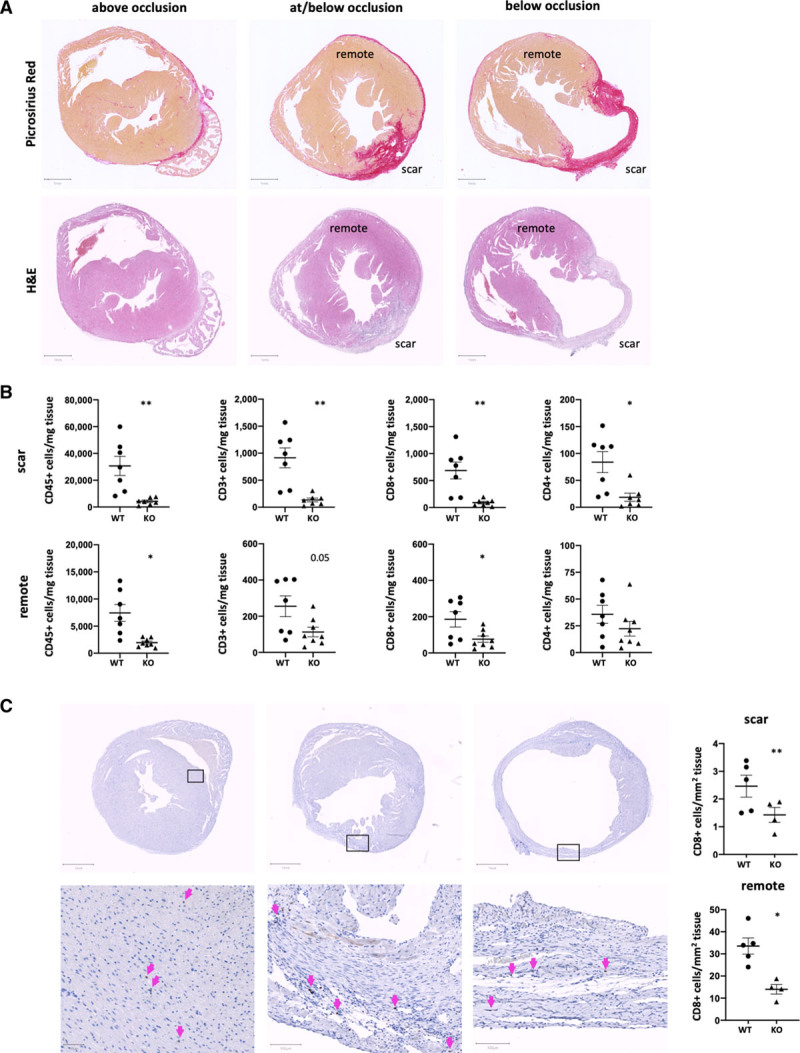
**Accumulation of cytotoxic CD8^+^ T cells is reduced in both scarred and remote myocardium in *Clec9a^−/−^* mice.** Type 1 myocardial infarction was induced in C57BL/6J (WT) and *Clec9a*^−/−^ C57BL/6J (KO) mice by permanent ligation of the left anterior descending artery. Hearts were collected 4 weeks after infarction and processed for histology and flow cytometry. **A**, Example micrographs showing Picrosirius red– and H&E-stained type 1 myocardial infarction heart sections above occlusion, approximately at the ligation point, and below the ligation point. **B**, Flow cytometry on single cell preparations of isolated scar and remote left ventricular tissue of the heart for quantification of CD3^+^, CD4^+^, and CD8^+^ cells/mg tissue. **C**, Immunohistochemistry staining for and quantifying numbers of CD8^+^ cells per mm^2^ scar and remote tissue section, respectively. Symbols represent individual mice. Error bars show mean±SEM; *P<0.05; **P<0.001 (1-tailed Student *t* test with Welch correction). H&E indicates hematoxylin and eosin; KO, knockout; and WT wild type.

To further confirm the relevance of cross-priming DC in the recruitment of myocardial T cells into the healthy remote myocardium, we induced T1MI by surgical left anterior descending artery ligation in both *Clec9a*^−/−^ and WT mice. This model generates myocardial injury with clearly separated scar and remote areas, allowing these to be analyzed independently (Figure 7A). Single cell suspensions were generated from dissected scar and remote LV tissue and analyzed for the presence of T cells 4 weeks after infarction. While there are clearly overall higher CD45^+^ immune cell numbers in the scarred compared with the remote tissue, a striking reduction is apparent in *Clec9a*^−/−^ compared with WT mice in both tissue areas (Figure 7B). Importantly, cytotoxic CD8^+^ T cell numbers are also clearly decreased in both areas in *Clec9a*^−/−^ mice, while changes in CD4^+^ T cell numbers are less prominent (Figure 7B).

Using immunohistochemistry to visualize CD8^+^ cells in situ, we confirmed their presence and decrease in both scarred and remote areas (Figure 7C). As evident from histology and similar to the observations previously noted, the total number of CD8^+^ cytotoxic T cells per square millimeter of tissue was considerably (≈10-fold) higher in the scar compared with the remote tissue, yet the reduction in numbers between Clec9a^−/−^ and WT mice was seen in both areas. This is also in line with the reduction in myocardial CD8^+^ T cells in Clec9a^−/−^ mice the isoproterenol-induced T2MI model (Figure IX in the Data Supplement), where healthy (“remote”) and damaged areas are interlaced making separate analysis challenging. Notably, numbers obtained from total LV tissue of isoproterenol-treated T2MI hearts fall in between scar and remote tissue of T1MI hearts, supporting that T2MI tissue is a mix of these 2 states. These results confirm that the depletion of DC cross-priming function blocks cytotoxic T cell infiltration and further show that this is not only true for tissue damaged during the initial infarction (scar), but also for supposedly healthy remote tissue indicating autoreactive cytotoxic T cells infiltrating the healthy myocardium.

## Discussion

In this study we show a role of DC cross-priming of cytotoxic CD8^+^ T cells in the persistence of myocardial immunopathology and the degree of fibrosis after ischemic injury, resulting in exacerbation of adverse LV remodeling and decline in cardiac function.

Anti-heart autoreactivity of the adaptive immune system has been implicated in structural remodeling, functional decline, and the development of heart failure.^21,25,53^ Here we define a mechanism by showing that cross-priming cDC1 exacerbate immune-mediated damage after ischemic injury of the heart. Depletion of the C-type lectin-like receptor CLEC9A/DNGR-1/CD370 blocks the cross-priming function of cDC1 and prevents the activation of cytotoxic CD8^+^ T cells likely to target cardiac antigens, thus preventing persistent immune-mediated damage.

In the cardiac context, DC ablation studies have previously yielded inconsistent results. Depletion of CD11c^+^ cells in a mouse model of acute MI led to deterioration of LV function and remodeling,^24^ while a recent study depleting DC via the transcription factor *Zbtb46* reduced infarct size, improved systolic function, and reduced CD3^+^ T cell numbers in the ischemic tissue.^25^ Human data are scarce and largely based on the presence or absence of DC in selected tissues. Myocardial DC activation was decreased in human dilated cardiomyopathy,^54^ while decreased circulating DC numbers correlated with decreased EF and increased LV dilation in chronic HF patients.^55^ This highlights a common complication in interpreting immunologic studies; broad immune cell populations are not homogenous entities, but complex interacting networks of subpopulations and factors with pleiotropic effects depending on the specific environment and varying functional characteristics of subpopulations are important.

The DC population in the heart is heterogenous already at healthy baseline conditions and includes a significant number of cross-priming DC, which are specialized in the recognition of necrotic cells.^56^ After tissue injury, they take up local antigens, migrate to draining lymph nodes and present processed antigens not only to CD4^+^, but also to cytotoxic CD8^+^ T cells. Cross-presenting DC may thus be central in inducing and boosting post-MI autoimmunity and directing T cell–mediated damage through autoreactive cytotoxic CD8^+^ T cells. While recent interest in the role of CD4^+^ helper T cells and their subpopulations has uncovered both detrimental and beneficial effects in heart disease,^57,58^ less is known about the role of cytotoxic CD8^+^ T cells after ischemic injury in the heart. Their pathogenic function in viral myocarditis is established,^59^ and they are recruited to other tissues undergoing ischemic injury including the brain.^60^ CD8^+^ T cells isolated from rats with experimental MI directly killed healthy cardiomyocytes in vitro^17^ and a recent study confirms detrimental inflammatory activity after MI.^18^ Notably, cytotoxic effects were MHC-dependent and antigen-specific, as cytotoxicity was significantly higher when using syngeneic CD8^+^ T cells from the same rat strain.^17^ In contrast, CD8^+^ T cells expressing the AT2R (angiotensin type 2 receptor) were found to infiltrate the tissue surrounding the infarct zone in another rat MI model and downregulate expression of proinflammatory cytokines, suggesting a potential cardioprotective role of this subset.^61^ The current study documents a dominant CD8^+^ T cell population in the heart, both after ischemic damage in mice as well as in human HF. Importantly, these T cells are not only observed as part of the total immune cell infiltrate in damaged areas but are also present in remote tissue that appears healthy. This is a crucial indicator that these T cells did not infiltrate in response to the initial infarction, but rather that they accumulated in the healthy myocardium in response to myocardial antigen. Over time, their cytotoxic activity against cardiomyocytes will contribute to the postinfarction pathologic burden on the heart. Depletion of DC cross-priming function blocked accumulation and activation of CD8^+^ T cells correlating with decreased postischemic immunopathology and functional decline.

Because of their unique position as T cell inducers, DC are a well-established pathogenic factor in prototype autoimmune diseases, including type 1 diabetes and rheumatoid arthritis,^62^ and several attempts have been made to tolerize DC for immunomodulatory therapies in autoimmune conditions including post-MI autoimmunity.^24^ Identification of cross-priming DC-mediated activation of anti-heart autoreactivity leading to exacerbation of adverse remodeling toward HF identifies a novel pathogenic entity leading to immune-mediated LV dilation. Blockade of this response offers a promising therapeutic prospect for immunomodulation post-MI and during HF.

## Acknowledgments

Author contributions: conceptualization: S.S.; methodology: S.S., M.B.F., M.G.H.; validation: S.S., M.B.F., M.G.H., E.F.; formal analysis: B.P., E.F., D.A.S., A.S., A.P., H.K., M.A., T.D., M.B.F., S.S.; investigation: B.P., E.F., A.S., C. J., A.P., H.K., M.A., T.D., J.B., M.B., M.B.F., S.S.; resources: M.D.S., SEH., N.R., M.B.F., M.G.H., S.S.; data curation: E.F., D.A.S.; writing (original draft): B.P., E.F., S.S.; writing (review and editing): B.P., E.F., M.G.H., F.S.N., S.S.; visualization: E.F., M.A., S.S.; supervision: R.C., D.A.S., F.S.N., M.B.F., M.G.H., M.D.S., N.R., S.S.; project administration: S.S.; and funding acquisition: S.S., F.S.N., R.C., SEH., N.R., M.D.S. We are grateful to Prof Caetano Reis e Sousa, The Cricks Institute London, for critical discussion and for providing Clec9a^−/−^ mice for pilot experiments. We would like to thank staff at the animal facility at Imperial College London and The Jackson Laboratories for help with animal husbandry and maintenance. We gratefully acknowledge the contribution of Stephen Rothery and the Facility for Imaging and Light Microscopy (FILM) at Imperial College London, Elaine Bechtel and the Histology and Light Microscopy Scientific Services, and William Schott at the Flow Cytometry Service at The Jackson Laboratory for expert assistance with the work described in this publication. We are grateful for technical advice and support from Michael M. Mclellan (The Jackson Laboratory). This study was supported by the supply of human tissue samples from the Cardiovascular Research Center Biobank at the Royal Brompton and Harefield National Health Service Foundation Trust (Research Ethics Committee approval: 09/H0504/104+5; Biobank approval number: NP001-06-2015) and National Health Service Blood and Transplant (Research Ethics Committee approval: 16/LO/1568). Informed consent was obtained from each patient involved in this study. We thank the patients for their kind donations.

## Sources of Funding

This work was generously supported by the British Heart Foundation (PG/16/93/32345 to S.S.; PG/16/17/32069 to R.C.; RM/17/1/33377 to S.E.H.; and CH/08/002/29257 and RG/15/1/31165 to M.D.S.), the Medical Research Council (via King’s College London) United Kingdom Regenerative Medicine Platform Immunomodulation Hub (MR/L022699/1 to S.E.H.), the National Institute for Health Research Imperial Biomedical Research Center (to F.S.N.) and the Leducq Foundation: Trans-Atlantic Networks of Excellence in Cardiovascular Research (to N.R.).

## Disclosures

None.

## Supplemental Materials

Expanded Methods and Materials

Data Supplement Figures I–IX

## Supplementary Material


