# Supplementary material for: Cross-Priming Dendritic Cells Exacerbate Immunopathology After Ischemic Tissue Damage in the Heart
Source: Circulation. 2020 Dec 10;143(8):821–36. doi: 10.1161/CIRCULATIONAHA.120.044581 (PMC7899721; doi:10.1161/CIRCULATIONAHA.120.044581)
Supplement: Supplementary file 1 [file cir-143-821-s001.pdf]

**SUPPLEMENTAL MATERIAL**

# Cross-priming dendritic cells exacerbate immunopathology after ischemic tissue damage in the heart.

Short title: Cross-priming DC and the post-MI heart.

Elvira Forte, PhD[1*], Bryant Perkins, BS[1*], Amalia Sintou, MS[2], Harkaran S. Kalkat, MBBS BS[2], Angelos Papanikolaou, BS[2], Catherine Jenkins, MS[2], Mashael Alsubaie, MS[2], Rasheda A. Chowdhury, PhD[2], Theodore M. Duffy, BS[1], Daniel A. Skelly, PhD[1], Jane Branca[1], Mohamed Bellahcene, PhD[2], Michael D. Schneider, MD[2], Sian E. Harding, PhD[2], Milena B. Furtado, PhD [1,3], Fu Siong Ng, MRCP PhD[2], Muneer G. Hasham, PhD[1], Nadia Rosenthal, PhD[1,2], Susanne Sattler, PhD[2&].

* E.F. and B.P. contributed equally to this work

Affiliations:

[1] National Heart and Lung Institute, Imperial College London, London, W12 0NN, UK

[2] The Jackson Laboratory, 600 Main Street, Bar Harbor, ME 04609, USA

[3] Amgen Biotechnology, Thousand Oaks, CA 91320-1799, USA

# Expanded Methods

**Mice:** All animal procedures carried out at Imperial College London were approved by the Imperial College Governance Board for Animal Research and in accordance with the UK Home Office Animals (Scientific Procedures) Act 1986 and Directive 2010/63/EU of the European Parliament on the protection of animals used for scientific purposes. All animal work carried out at The Jackson Laboratory was approved by The Jackson Laboratory Institutional Animal Care and Use Committee and were in accordance with national and international guidelines and regulations. Mice used were 8- to 12-week-old males and females; control mice were age- and sex-matched littermates. Mice were housed under SPF conditions (Imperial College London) or in conventional cages (The Jackson Laboratories) in temperature-controlled facilities on a 12h light/dark cycle on standard diet and bedding (ECO2). Mouse strains used in this study were B6(Cg)-Clec9at1.1Crs/J (CLEC9a$^{-/-}$), B6.129S2-Cd8atm1Mak/J (*CD8-/-*) and C57BL/6J (WT). All are available commercially from The Jackson Laboratory. The B6(Cg)-Clec9at1.1Crs/J mouse strain was maintained in heterozygous sibling mating pairs to generate wild type, heterozygous and homozygous littermates. Mice were genotyped using a standard thermocycler profile and the following set of primers: 5'-TGT AGA CTG CTT CAC CAC TCC-3', 5'- TGC ATC AGC TGA AGA CAT TTT T -3', and 5'- GCC GTT CTT CTG CTT GTC G -3'.

***Experimental planning and statistical analysis:*** Experimental design of *in vivo* mouse studies was guided by the recommendations of the NC3R ARRIVE guidelines[40]. After acclimatization, mice were randomly allocated to experimental and control groups by drawing from a joint pool of age- and sex-matched mice bred-in house or purchased from Charles River, UK,. Husbandry and care was provided by blinded animal facility staff, and analysis was performed by a blinded researcher. During experimentation on the life animal (e.g. echocardiography) this was achieved through

concealment of cage labels and assigning a separate set of numbers for *ex vivo* analyses (e.g. immunofluorescence, IHC). Experimental group sizes were defined based on effect sizes of the primary outcome measure achieved in a previous study designed to establish the T2MI model[31] with the aim to achieve 95% power, with 0.05 set as alpha error. Statistical analysis was performed using SPSS or GraphPad Prism 6 and data were presented as mean±s.e.m throughout. Normal distribution of parametric data was tested using Shapiro-Wilk test. Comparison between 2 groups was performed using student's t-test. Comparison between multiple experimental groups was performed using one- or two-way ANOVA with multiple comparisons post hoc tests to obtain multiplicity-adjusted p-values. Differences were considered significant at $p<0.05$.

***Myocardial infarction:*** MI was induced as previously described[30] in male and female mice 10-12-weeks of age. Briefly, the left anterior descending branch of the coronary artery (LAD) was permanently ligated above branching, about 1mm below the tip of the left auricle. Only mice with > 30% left ventricular ischemia were included in this study. The mouse thoracic cavity was then closed and sutured. Sustained release 0.05 µg/g buprenorphine gel formulation was administered subcutaneously at the time of surgery for analgesia. Bupivacaine was administered locally to chest suture site. Mice were individually monitored daily for the first 7 days, followed by weekly monitoring until the endpoint of experiments. A total of 39 mice were used for LAD ligation surgery in this study. Mortality rate was 28%. 3 animals were excluded from analysis due to unsatisfactory (>30% LV scar) MI induction.

***Isoproterenol treatment:*** Mice were treated with isoproterenol HCL (Sigma-Aldrich, St. Louis, MO, USA) in Dulbecco's phosphate-buffered-saline (DPBS; Sigma-Aldrich) by a single intraperitoneal injection at a dose of 160 mg/kg as established previously as T2MI model[31].

Control mice were treated with DPBS at equivalent volume. Tissue was generally harvested either 1, 2 or 4 weeks post-injection. On the day of tissue collection 200 ul of blood was collected. Blood was incubated on ice for 30 minutes, then centrifuged 3 minutes at max rcf. Serum was collected and stored at -20 ºC for later use. Heart and lymphoid tissue were isolated after *in situ* perfusion with ice-cold DPBS supplemented with 0.9 mM $CaCl_2$ through the apex of the left ventricle of the heart to clear blood from heart chambers and blood vessels. A total of 194 mice were used for T2MI experiments. Mortality rate was >5%.

***Langendorff perfusion and optical mapping:*** Experiments were performed as previously[37,38]. Briefly, explanted hearts were perfused and superfused with modified Tyrode's solution (in mM: 130 NaCl, 5.4 KCl, 0.3 $NaH_2PO_4$, 1 $MgCl_2$, 10 HEPES, 18 $CaCl_2$, and 10 D-Glucose, [pH 7.4]) Electrocardiograms (ECG) were recorded during all experiments using LabChart 8 software in conjunction with Powerlab and BioAmp systems (ADInstruments Pty Ltd, UK) and programmed electrical stimulation (PES) protocols were carried out via the pacing electrode at the left ventricle using a stimulator (MicroPace, USA). For optical mapping of transmembrane voltage, an excitation-contraction uncoupler, blebbistatin (5µM), and voltage sensitive dye, Di-4-ANNEPS (5µM) were administered to the heart and signals recorded using complimentary metal-oxide semiconductor (CMOS) cameras (RedShirtImaging LLC, USA). ECG recordings and optical recordings were analysed using a method previously described[37,38].

***Echocardiography:*** Transthoracic echocardiography was performed by a blinded operator using a high-frequency ultrasound system Vevo 770 (VisualSonics Inc., Toronto, Canada) with a 30-MHz linear transducer. Mice were anesthetized with 1-2 % isoflurane and heart rate

maintained at 450 ± 50 beats/minute. Warmed ultrasound gel and a heating pad were used to maintain body temperature at 37 ± 0.5 ºC. Left ventricular (LV) parameters were obtained from 3 M-mode traces at base, mid-level and apex from both the parasternal long- and short-axis view. Images were analyzed using the Vevo 770 workstation software. Analysis was performed at baseline, week 2, 4 and 6 after isoproterenol-induced ischemia to evaluate left ventricular function and chamber dimensions.

***Human tissue samples:*** All work was carried out under the Human Tissue Act 2004 and conformed with the principles of the Declaration of Helsinki. Tissue and clinical data were fully anonymized before handing to researchers. Human LV tissue was obtained from hearts of end-stage heart failure patients removed during transplant surgery (REC: 09/H0504/104+5; Biobank approval number: NP001-06-2015). Donor hearts unsuitable for transplantation were used as controls (REC: 16/LO/1568). Informed consent was obtained from patients or next of kin. Patient characteristics: ischemic heart disease; 2 females (45, 60 years), 5 males (57-67 years); myocarditis; 2 females (20, 28 years), 1 male (51 years); donors; 3 females (17, 38, 50 years), 2 males (48, 39 years).

***Immunofluorescence staining:*** For detection of $CD3^+$ and $CD8^+$ T cells in heart failure hearts, frozen sections of human heart tissue were stained with mouse anti-human CD3 (clone UCHT1 at 1:100) and mouse anti-human CD8 (clone HIT8a at 1:100) with Alexa Fluor® 488 goat anti-mouse IgG (poly4053 at 1:500) (all purchased from BioLegend, London, UK). Wheat germ agglutinin (WGA)-TexasRed (Invitrogen, Thermo Fisher Scientific, Rochford, UK) was used as membrane counterstain. Sections were mounted using DAPI mounting medium

(Abcam, Cambridge, UK). Images were captured using a Zeiss Axio Observer inverted microscope and processed using the public domain software ImageJ[35].

**Single cell sequencing:** Single cell RNA sequencing was performed as part of a previous study[29] using the 10xChromium platform with version 2 chemistry. For this study, raw Illumina data were processed using CellRanger 3.0 (10x Genomics); data quality control, normalization, clustering, and marker analysis were performed using Seurat software package version 3.0[32] in R version 3.6.2[33]. Data from seven samples (homeostasis, and day 1, 3, 5, 7, 14, 28 post-MI) were merged, after filtering out cells in which <200 or >5,000 genes were detected; >20% of reads were derived from mitochondria; or >15,000 UMIs ('nCount_RNA') were sequenced (based on the visualization of QC matrix as violin plots as per standard procedure). A single Seurat object with 51,687 cells was obtained. Expression of 31126 transcripts was quantified. Read counts were normalized using log transformation. Cell cycle stage was estimated and regressed out using methods implemented in Seurat (and detailed at https://satijalab.org/seurat/v3.0/cell_cycle_vignette.html). In addition to cell cycle stage, the number of genes detected, number of UMIs detected, and percentage of mitochondrial reads were also regressed out. Variable genes were detected using the Seurat function FindVariableFeatures with default parameters (selection method 'vst', nfeatures 2000). Dimensionality reduction was used to explore transcriptional heterogeneity and for clustering. Specifically, PCA and UMAP were used for dimensionality reduction. The first 32 principal components (PCs) were used as input for the "RunUMAP" function, 25 PCs for "FindNeighbours" and resolution 0.5 to "FindClusters". 25 different clusters were obtained and merged into 11 main populations for the purpose of this study. 897 DC were subset and further subclustered using 16 PC for "RunUMAP" and FindNeighbours" and resolution 0.5 to

"FindClusters". 1040 NKT cells were subsetted and further sub-clustered using 13 PC for "RunUMAP" and FindNeighbours" and resolution 0.5 to "FindClusters". Cells with *Cd3g* expression>0.1 were subset to quantify $Cd4^{+}$ and $Cd8^{+}$ fractions. Two Seurat functions were employed to identify marker genes: FindAllMarkers() and FindMarkers(). DotPlot(), FeaturePlot() and DoHeatmap() functions were used for plotting gene expression data. All sequencing data are available in the Array Express repository under the accession number EMTAB-7895.

***Histology and Immunohistochemistry:*** Hearts of treated and untreated mice were excised after perfusion as described above and fixed in 10% neutral buffered formalin overnight and stored in 70% ethanol. For wax-embedding and histology, tissue samples were dehydrated in an increasing gradient of ethanol and embedded in paraffin. Five µm sections were cut and de-waxed and rehydrated in an ethanol gradient. Sections were stained with hematoxylin and eosin (H&E) and Picrosirius Red. All reagents were purchased from Sigma Aldrich (Sigma-Aldrich, Dorset, UK). Semi-quantitative scoring was performed as established previously[34]. For CD8 immunohistochemistry sections were stained after 20 minutes of citrate buffer antigen retrieval with rat anti-mouse CD8 (Invitrogen, Thermo Fisher Scientific, Rochford, UK, #14-0808-80 at 1:1000) and rabbit anti-rat-HRP (Vector laboratories, Peterborough, UK, #AI-4001 at 1:50) with hematoxylin as nuclei counterstain. Images were captured using a Hamamatsu NanoZoomer 2.0 slide scanner (Hamamatsu, San Jose, CA, US) or a LMD7000 microscope (Leica microsystems, Milton Keynes, UK) and processed for quantification of nuclei (cell count) and area of fibrosis using NDP.view2 Plus Image viewing software (Hamamatsu, San Jose, CA, US) and the public domain software ImageJ (NIH; http://rsb.info.nih.gov)[35].

Quantification of DAB+ CD8 T cells was performed using QuPath open source bioimage analysis software (https://qupath.readthedocs.io/en/latest/index.html#)[36].

**Scoring of damage parameters:** Hematoxylin & eosin stained sections were used to analyze mononuclear cell infiltration. Picrosirius Red staining was used to analyze and score fibrosis. Individual parameters were scored on a scale of 0 (none), 1 (mild), 2 (moderate) to 3 (severe) as described previously[34]. Scores were obtained from 4 areas each on four midline cross sections per animal by a blinded researcher. Images were captured using a LMD7000 microscope (Leica microsystems, Milton Keynes, UK) and processed for quantification of nuclei and area of fibrosis using the public domain software ImageJ (NIH; http://rsb.info.nih.gov)[35].

***Cardiac Troponin I ELISA:*** To determine cardiac troponin I (cTnI) concentrations in serum, a mouse cTnI ELISA Kit (MyBioSource, San Diego, CA, USA) was used according to the manufacturer's instructions. Serum was diluted 2-fold in supplied diluent. Optical densities were measured at 450 nm using a SpectraMAX i3 microplate reader (Molecular Devices, San Jose, CA, USA). A standard curve was created to calculate cTI concentrations in pg/ml from optical densities.

***Immune cell isolation from the heart and lymphoid tissues:*** To generate single cell cardiac suspensions for flow cytometry, a modified digestion protocol was used as previously described[39]. Hearts were excised, chopped 30 times with surgical scissors and placed in 3 ml digestion buffer. For T1MI experiments, LV tissue was separated into scar and remote tissue. Digestion buffer consisted of 2 mg/ml collagenase type IV (Worthington Biochemical

Corporation, Lakewood, NJ, USA), 1.2 U/ml dispase II (ThermoFisher Scientific, Waltham, MA, USA), and 16.6 ul/ml precision count beads (BioLegend, San Diego, CA, USA) in DPBS supplemented with 0.9 mM $CaCl_2$. Tissue was incubated at 37ºC for 15 minutes. After incubation, tissue was triturated 12 times. Tissue was incubated and triturated 2 more times for a total of 45 minutes. The final trituration was increased to 30. Tissue was filtered through sterile cheesecloth and washed with ice-cold DPBS supplemented with 0.9 mM $CaCl_2$ for 20 minutes at 200 rcf at 4 ºC. To generate single cell lymphoid suspensions, excised spleen and mediastinal lymph nodes were incubated at 37ºC for 30 minutes in 3 ml and 0.5 ml digestion buffer, respectively. Lymphoid digestion buffer consists of 0.24 U/mg, 400 Mandl U/ml Collagenase D (Roche Diagnostics GmbH Mannheim, Germany) in FBS free DMEM. Cells were washed twice in FACS buffer supplemented with 2% FBS spun for 5 minutes at 400 rcf at 4 ºC. Splenocytes were resuspended in FACS buffer supplemented with 2% FBS spun at $10^8$ cells/ml for antibody staining. All mediastinal lymphocytes were resuspended in FACS buffer supplemented with 2% FBS for antibody staining.

***Flow cytometry:*** Cell suspensions were stained with of anti-mouse CD45 (CD45-SB600, eBioscience (San Diego, CA, USA), cat# 63-0451-82, clone 30-F11, which reacts with all isoforms and both CD45.1 and CD45.2 alloantigens, 1:40), anti-mouse CD3e (CD3e-PerCP-Cy5.5, BD Bioscience, cat# 551183, clone 145-2C11, 1:20), anti-mouse CD4 (CD4-BV650, BD Bioscience, cat# 563747, clone RM4-5, 1:60), anti-mouse CD8a (CD8a-BV711, BD Bioscience, cat# 563046, clone 53-6.72, 1:20), anti-mouse CD62L (CD62L-FITC, BioLegend, cat# 104406, clone MEL-14, 1:40), anti-mouse CD44 (CD44-APC-Cy7, Tonbo Biosciences (San Diego, CA, USA), cat# 25-0441-U100, clone IM7.8, 1:20). DC staining consisted of anti-mouse CD45 (CD45-SB600, eBioscience, cat# 63-0451-82, clone 30-F11, which reacts with all isoforms and

both CD45.1 and CD45.2 alloantigens, 1:40), anti-mouse CD11c (CD11c-FITC, BioLegend, cat# 117306, clone N418, 1:40), anti-mouse CD11b (CD11b-PE-Cy7, BD Bioscience, cat# 552850, clone M1/70, 1:100), anti-mouse XCR1 (XCR1-APC, BioLegend, cat# 148206, clone ZET, 1:40), anti-mouse Ly6C (Ly6C-BV570, BioLegend, cat# 128029, clone HK1.4, 1:80), anti-mouse Ly6G (Ly6G-BV421, BD Bioscience, cat# 562737, clone iA8, 1:40), anti-mouse MHCII (MHCII-BV786, BioLegend, cat# 107645, clone M5/114, 1:40). All samples (50ul) were stained with 10 ul of antibody cocktail at room temperature for 30 minutes. Samples were washed with and resuspended in FACS buffer supplemented with 2% FBS. 10ul of 2 ug/ml DAPI stock solution was added to each 250ul sample. Samples were acquired using a BC FACSymphony A5 (Becton Dickinson) and analyzed using FlowJo 10.2 (Treestar, Ashland, OR, USA) software (www.flowjo.com). Fluorescence minus one (FMO) controls were used for gating on individual populations.

## Supplementary Figures

### Figure I

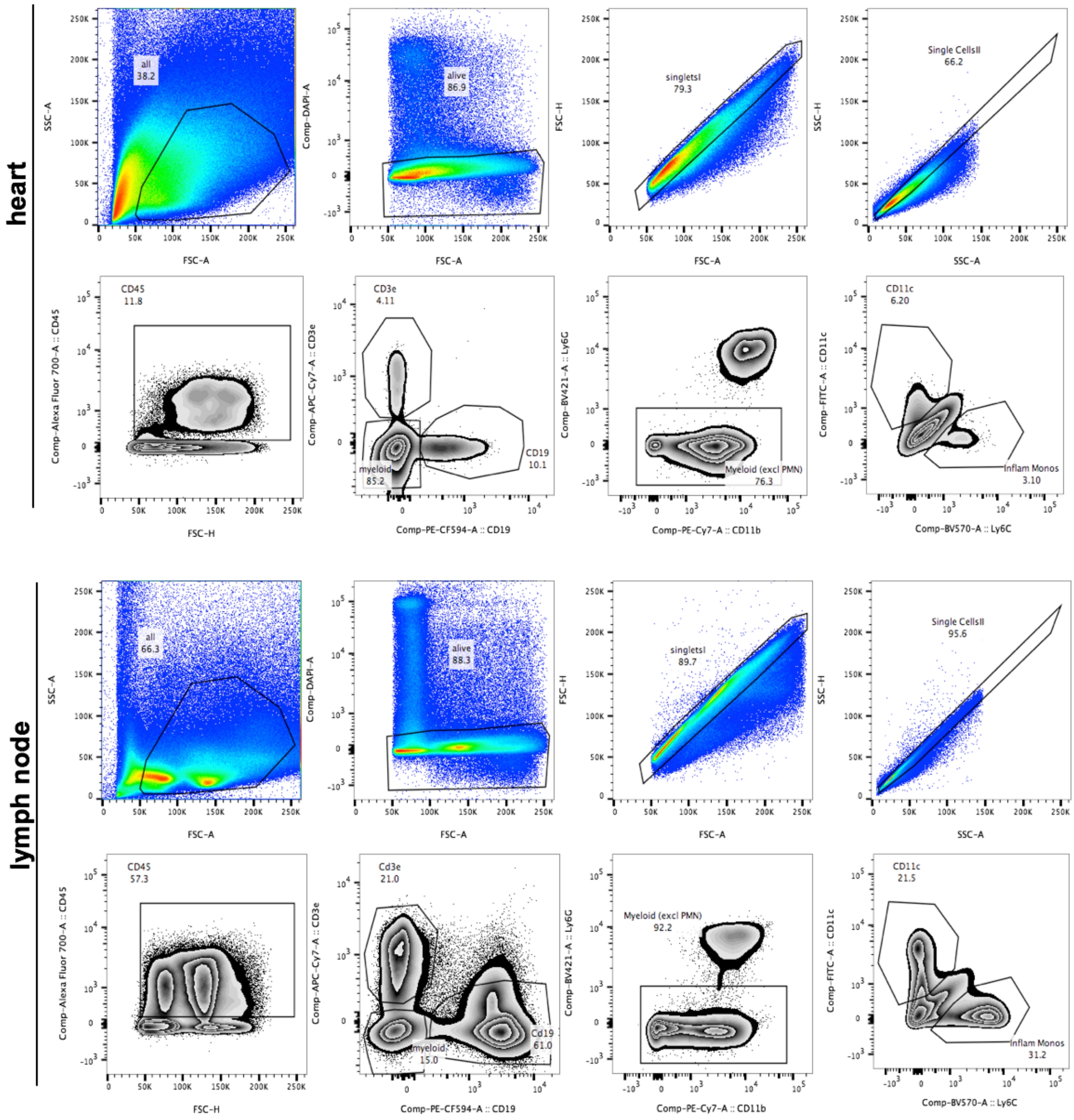


**Supplementary Figure I:** Flow cytometry gating strategy

**Figure II**

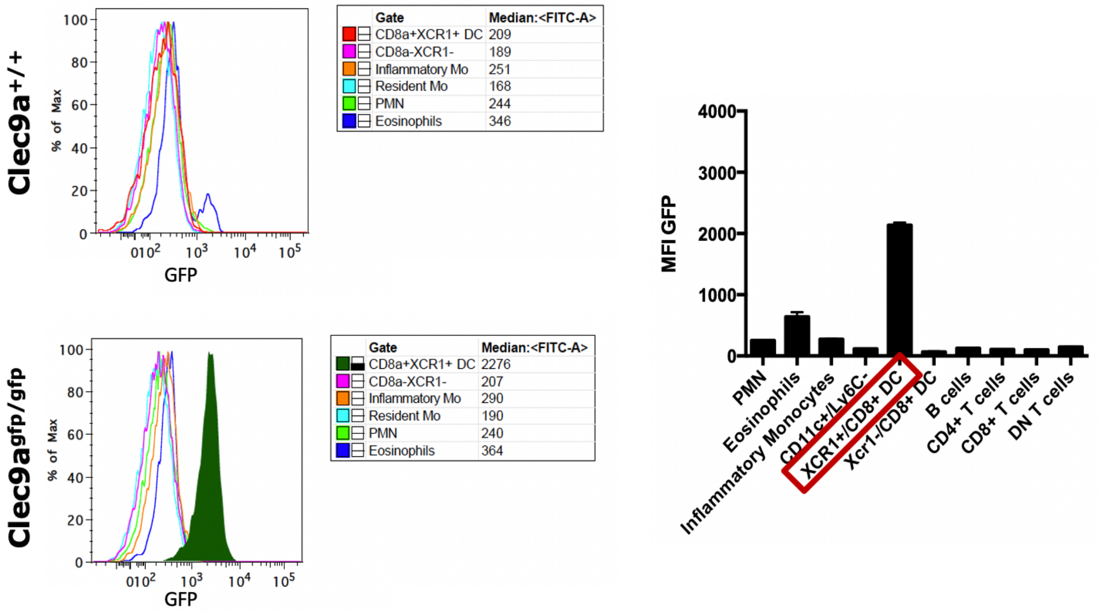


**Supplementary Figure II:** Specificity of *Clec9a* expression for the XCR1+ DC population

**Figure III**

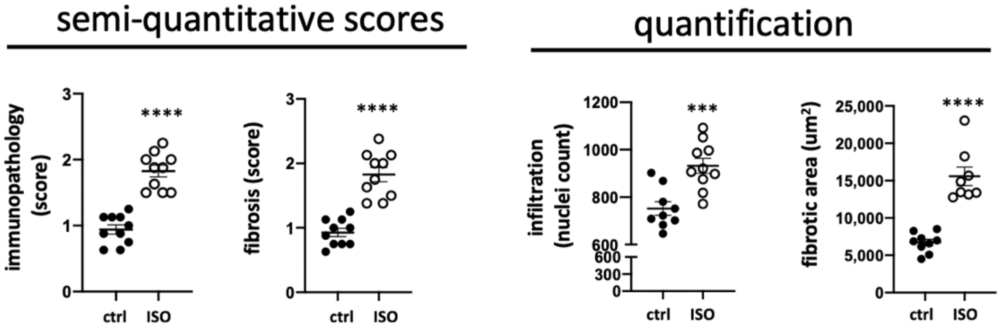


**Supplementary Figure III:** Comparison of semi-quantitative and quantitative histopathology readouts (immunopathology, fibrosis). Semi-quantitative scores presented in main figure 3. Symbols represent individual mice. Error bars show mean +/- s.e.m., ***p<0.0001, ****p<0.00001 (two-tailed Student's t-test with Welch correction).

**Figure IV**

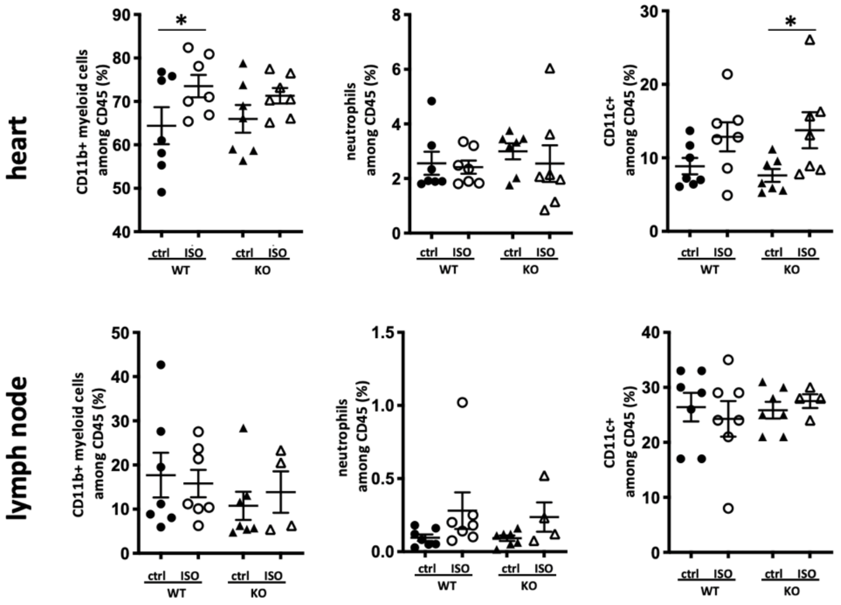


**Supplementary Figure IV:** $CD11b^+$ myeloid cells, $CD11b^+Ly6c^+$ neutrophils and $CD11c^+$ DC in baseline and isoproterenol-treated (1 week) WT and *Clec9a*$^{-/-}$ mice. Symbols represent individual mice. Error bars show mean +/- s.e.m., *p<0.05 (two-way ANOVA with Sidak's multiple comparisons post hoc test for multiplicity-adjusted p-values).

**Figure V**

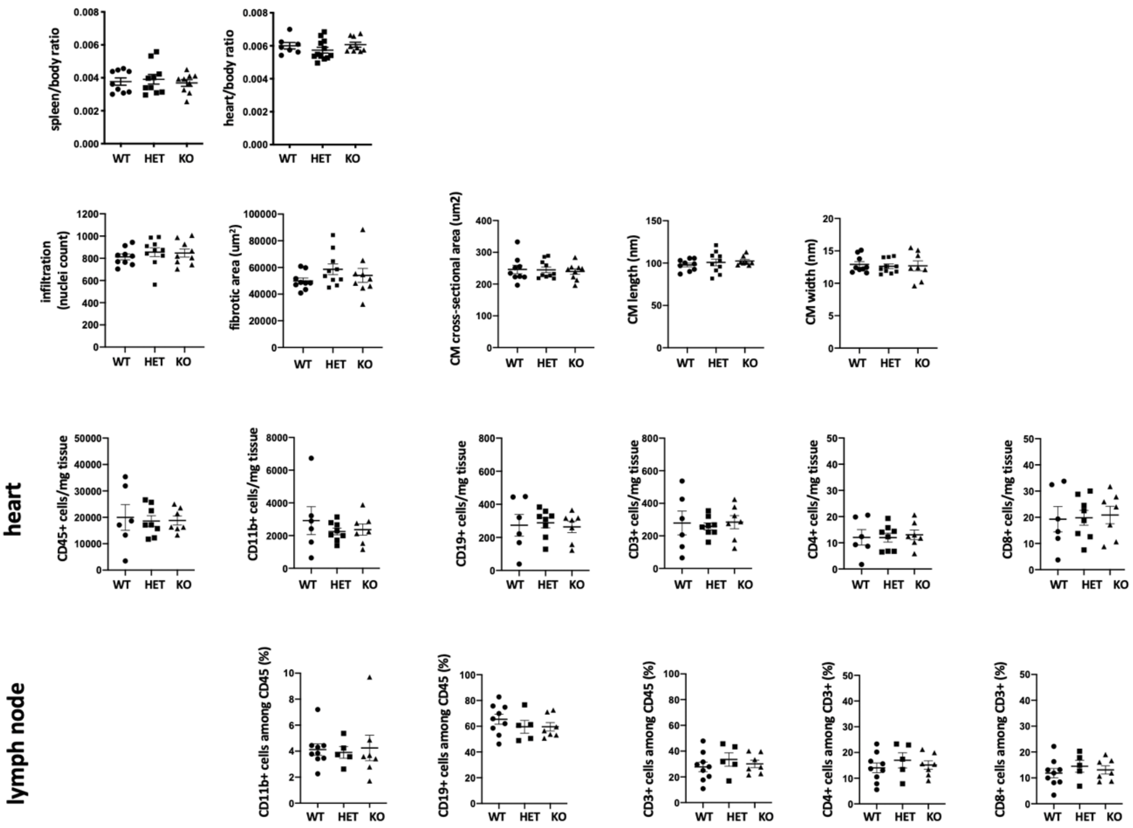


**Supplementary Figure V:** Baseline morphological and immunological parameters in WT and *Clec9a*$^{-/-}$ hearts and lymph nodes. Symbols represent individual mice. Error bars show mean +/- s.e.m., statistics: one-way ANOVA with Dunnett's multiple comparisons post hoc test for multiplicity-adjusted p-values.

**Figure VI**

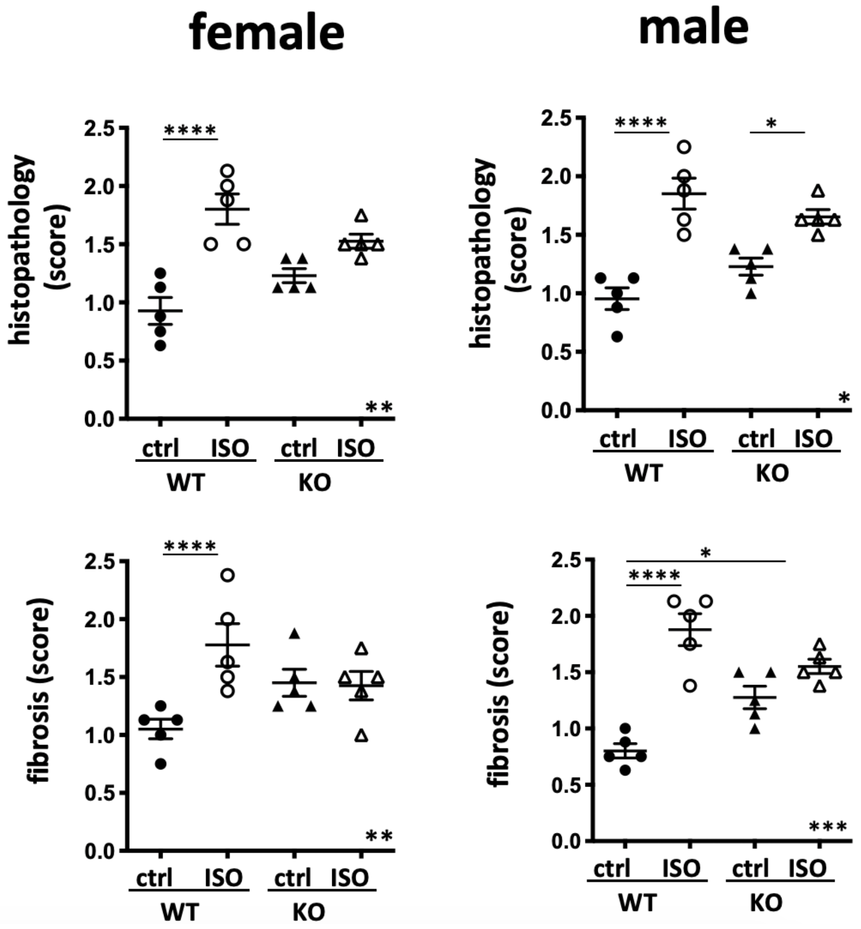


**Supplementary Figure VI:** Comparison of histopathology readouts (immunopathology, fibrosis) between male and female mice. Symbols represent individual mice. Error bars show mean +/- s.e.m., *p<0.05, ****p<0.00001 (two-way ANOVA with Sidak's multiple comparisons post hoc test for multiplicity-adjusted p-values).

**Figure VII**

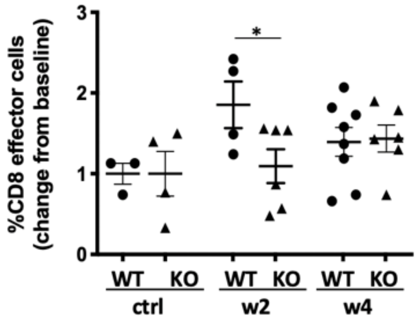


**Supplementary Figure VII:** CD8 effector T cells in LV tissue of isoproterenol-treated WT and *Clec9a*$^{-/-}$ mice week 2 and 4 after challenge. Symbols represent individual mice. Error bars show mean +/- s.e.m., *p<0.05 (two-way ANOVA with Sidak's multiple comparisons post hoc test for multiplicity-adjusted p-values).

**Figure VIII**

A

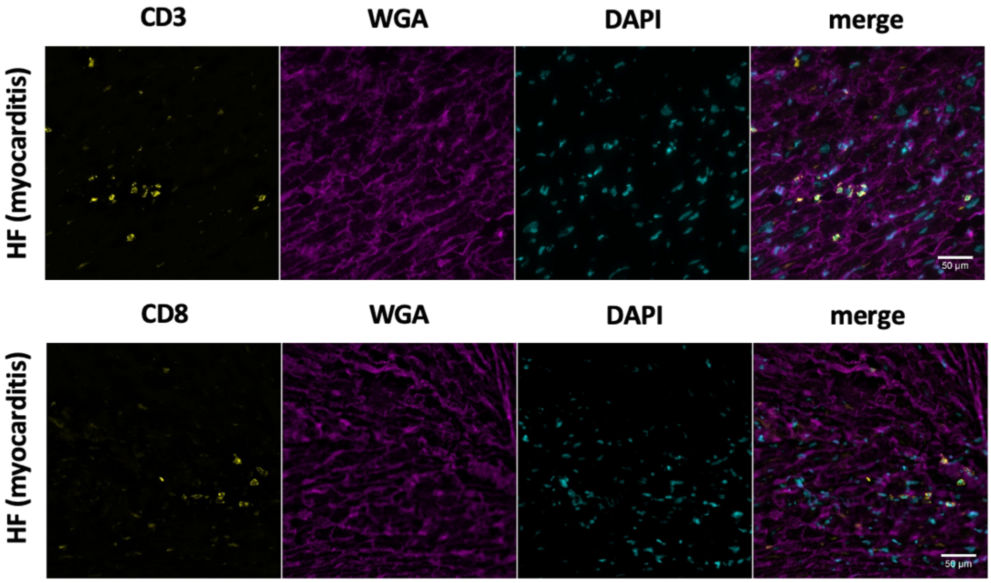


B

| ID | Male | Age | HF trigger | CD3+ cells/cm2 | CD8+ cells/cm2 | CD4+ cells/cm2 |
|---|---|---|---|---|---|---|
| 1 | | 20 | Myocarditis | 156 | 98 | 23 |
| 2 | | 28 | Myocarditis | 289 | 127 | 0 |
| 3 | x | 51 | Myocarditis | 111 | 77 | 0 |
| | | | mean +/-sem | 186 +/- 54 | 101 +/- 14 | 8 +/- 8 |

**Supplementary Figure VIII:** Human LV tissue was obtained from end-stage HF patients due to myocarditis during transplant surgery. (A) Representative images of the number of $CD3^+$ and $CD8^+$ T cells / $cm^2$ heart tissue section. (B) Patient information.

**Figure IX**

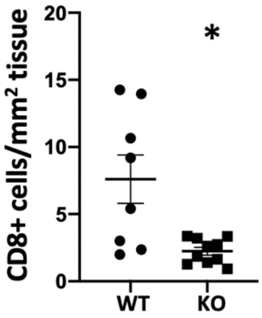


**Supplementary Figure IX:** Quantification of $CD8^+$ cells per $mm^2$ tissue section in isoproterenol-treated T2MI WT and *Clec9a*$^{-/-}$ mice 4 weeks after treatment. Symbols represent individual mice. Error bars show mean +/- s.e.m., *p<0.05 (two-tailed Student's t-test with Welch correction).
